# Missing heritability and missing Fst of candidate genes: why does gene variation differ from trait variation in trees?

**DOI:** 10.1186/1753-6561-5-S7-I1

**Published:** 2011-09-13

**Authors:** Antoine Kremer

**Affiliations:** 1 INRA, 69 Route d'Arcachon, Cestas 33610, France

## 

Molecular footprints of phenotypic variation are usually explored by two different approaches in trees. On the one hand, association genetics is seeking for statistical correlation between SNPs and traits owing to the significant heritability that is usually observed in progeny tests. On the other hand, detection of outlier Fst values of single SNPs has also been implemented to account for the very large differentiation of traits that are observed in provenance tests. The rationale of both approaches is based on the extensive within or between population genetic variability that has been widely recorded in fields tests. While the cataloguing of candidate genes has steadily increased in trees, so has the inventory of their diversity in natural or breeding populations. There is now a rapidly growing body of experimental results showing a very large discrepancy between the expectations of both approaches and the SNP variation that is monitored in candidate genes. Most association studies show that single SNPs explain usually less than 5% of the phenotypic variation of the trait, while Fst values are at best of the same value than neutral markers. In my presentation I will explore the reasons of the decoupling between trait and gene variation, by focusing on the multilocus structure of traits, as compared to the monolocus SNP information. Indeed, association studies and Fst outlier detection are essentially based on single locus approaches, while traits are multidimensional structures. There are at least three properties of multilocus structures that will be investigated: cumulativity, interactions and covariation of gene effects. I will show that cumulativity and interactions may explain the discrepancy in the case of association studies, while covariation of gene effects (at the between population level) explain the missing Fst of genes underlying adaptive traits. These conclusions are supported by experimental results, theoretical background and simulation predictions.

